# Plasma Phospholipids and Incident Ulcerative Colitis: A Prospective Cohort Study in the UK Biobank

**DOI:** 10.14309/ctg.0000000000001051

**Published:** 2026-06-04

**Authors:** Jialu Tang, Dawei Zhou, Shaojun Liu

**Affiliations:** 1 Department of Gastroenterology, The Third Xiangya Hospital, Central South University, Changsha, Hunan, China;; 2 Department of Infectious Diseases, The Third Xiangya Hospital, Central South University, Changsha, Hunan, China.

**Keywords:** ulcerative colitis, phospholipids in lipoprotein particles, incidence, UK biobank

## Abstract

**INTRODUCTION:**

To investigate the association between plasma levels of total phospholipids in lipoprotein particles and the risk of incident ulcerative colitis (UC).

**METHODS:**

This prospective cohort study analyzed data from 254,921 participants in the UK Biobank (2006–2020), excluding those with preexisting UC or missing data. Phospholipid levels were measured by nuclear magnetic resonance spectroscopy. Cox proportional hazards models were adjusted for demographic, lifestyle, socioeconomic, and genetic factors. Dose-response and subgroup analyses were conducted using generalized additive models.

**RESULTS:**

During a median follow-up duration of 15.78 years, 1,330 incident UC cases were identified. Higher phospholipid levels demonstrated a significant inverse association with UC risk. After full adjustment, each unit increase in phospholipids was associated with a 16% lower risk (hazard ratio 0.84, 95% confidence interval 0.75–0.95, *P* = 0.006). Quartile analyses showed lower risks in higher quartiles, with the risk reduction most evident by the third quartile and then generally stable (Q4 vs Q1: hazard ratio 0.83, 95% confidence interval 0.71–0.97, *P* = 0.020). Generalized additive and quartile analyses suggested a nonlinear plateau/threshold pattern rather than a strictly linear association. Subfraction analyses showed heterogeneous associations, with inverse patterns mainly observed for phospholipids carried by high-density lipoprotein cholesterol, intermediate-density lipoprotein, and low-density lipoprotein cholesterol particles, whereas chylomicron- and several very low-density lipoprotein-related phospholipids showed null or positive/nonprotective associations.

**DISCUSSION:**

Higher plasma total phospholipid levels are independently associated with reduced UC risk, suggesting phospholipid metabolism may play a protective role in UC pathogenesis. However, this association is not uniform across lipoprotein subfractions. These findings highlight potential metabolic biomarkers and mechanistic pathways requiring further validation for UC prevention.

## INTRODUCTION

Ulcerative colitis (UC) represents a chronic and debilitating inflammatory bowel disease characterized by relapsing and remitting inflammation of the colonic mucosa (1). Its etiology is complex (2), arising from a dysregulated immune response to intestinal microbiota in genetically susceptible individuals, further modulated by environmental and lifestyle factors (3). The global burden of UC is substantial and increasing (1), particularly in newly industrialized countries, leading to significant healthcare costs (4), impaired quality of life, and an elevated risk of colorectal cancer (5). Despite advances in treatment, a substantial proportion of patients do not achieve sustained remission with existing therapies, highlighting the critical need to identify novel, modifiable risk factors and protective agents that could inform preventive strategies and deepen our understanding of the disease's pathogenesis (6).

The role of lipids and systemic metabolism in immune regulation and intestinal inflammation has garnered increasing interest (7). Phospholipids (8), fundamental constituents of all cellular membranes and lipoprotein particles, are essential for maintaining intestinal barrier integrity, cellular signaling (9), and producing anti-inflammatory lipid mediators (10). They are crucial components of various lipoprotein classes, including chylomicrons, very low-density lipoprotein (VLDL), low-density lipoprotein cholesterol (LDL), and high-density lipoprotein cholesterol (HDL) (11), which are increasingly recognized not merely as passive lipid carriers but as active players in inflammatory and immune processes (12). However, the specific relationship between systemic levels of phospholipids—both total concentrations and their distribution across lipoprotein subfractions—and the risk of developing incident UC remains poorly characterized in large prospective cohorts.

To address this significant knowledge gap, we conducted a large-scale prospective cohort study using data from the UK Biobank. Our primary objective was to investigate the association between baseline plasma levels of total phospholipids in lipoprotein particles and the subsequent risk of developing incident UC. We further sought to elucidate the dose-response relationship using a generalized additive model. Finally, we also explored whether this association is consistent across key demographic and clinical subgroups. The aim of this comprehensive analysis was to determine whether phospholipids represent a novel and independent biomarker for UC risk, potentially illuminating new pathways for therapeutic intervention and prevention.

## METHODS

### Study population

**Figure 1.**
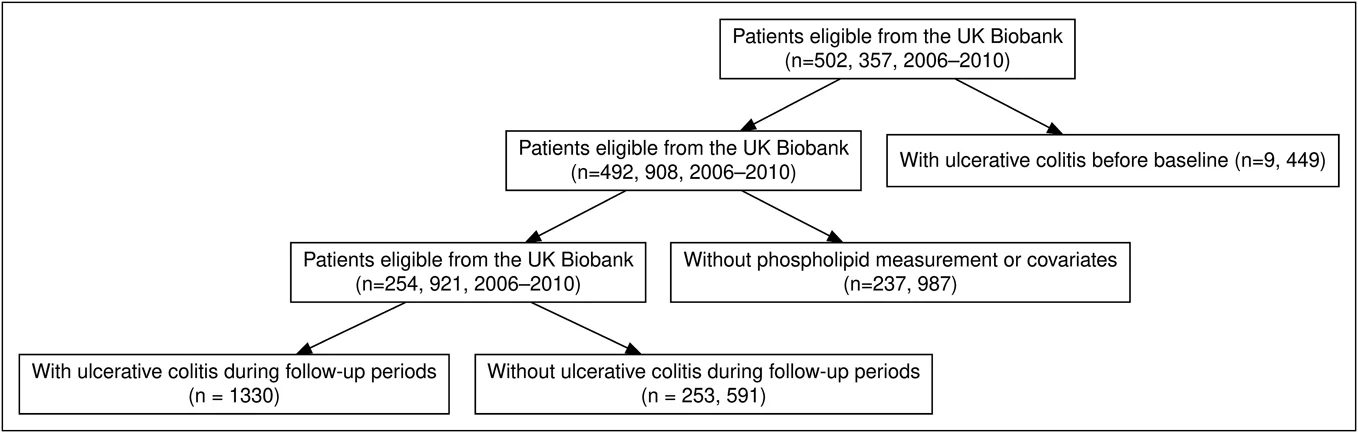
Flowchart of participant selection from the UK Biobank study. UK Biobank: United Kingdom Biobank.

This prospective cohort study used data from the UK Biobank, a large-scale biomedical database containing in-depth genetic and health information from over 500,000 participants aged 40–69 years recruited across the United Kingdom between 2006 and 2010. Potentially eligible participants were identified through National Health Service registers and invited to attend one of the 22 dedicated assessment centers situated throughout England, Scotland, and Wales. At baseline, participants completed comprehensive assessments, including self-completed touch-screen questionnaires, computer-assisted interviews, physical measurements, and the donation of biological samples. The study design and participant recruitment have been described in detail previously (13,14). For the present analysis, we excluded participants with a preexisting diagnosis of UC at baseline (n = 9,449) and those without available measurements of plasma phospholipids or key covariates (n = 237,987). The final analytical cohort consisted of 254,921 participants (Figure 1). The UK Biobank received ethical approval from the Northwest Multi-Centre Research Ethics Committee, and all participants provided written informed consent.

### Assessment of exposure and covariates

The primary exposure was plasma total phospholipids in lipoprotein particles, measured using nuclear magnetic resonance spectroscopy (Nightingale Health Platform). In addition to total phospholipids in lipoprotein particles, we quantitatively assessed phospholipid concentrations across specific lipoprotein subclasses and their relative composition using nuclear magnetic resonance spectroscopy (https://biobank.ndph.ox.ac.uk/ukb/label.cgi?id=220). The measured exposures included phospholipid contents in (i) major lipoprotein classes (Chylomicrons and Extremely Large Very Low-Density Lipoprotein, Very Large Very Low-Density Lipoprotein, Large Very Low-Density Lipoprotein, Medium Very Low-Density Lipoprotein, Small Very Low-Density Lipoprotein, Very Small Very Low-Density Lipoprotein; Intermediate-density lipoprotein; Low-density lipoprotein subclasses [Large, Medium, and Small Low-density lipoprotein]; High-density lipoprotein subclasses [Very Large, Large, Medium, and Small High-density lipoprotein]) and (ii) the relative percentage of phospholipids to total lipids within each of these specific subfractions. These measures provide detailed insights into the distribution and composition of phospholipids across the entire lipoprotein spectrum, allowing for the investigation of which specific particle subtypes may be most strongly associated with UC risk.

Covariates included demographic factors (age, sex, ethnicity), socioeconomic status (Townsend deprivation index), lifestyle factors (body mass index, smoking status, alcohol use, physical activity measured by metabolic equivalent of task min/wk, healthy eating index), and genetic risk factors of UC. The genetic risk score was calculated based on established UC-associated single nucleotide polymorphisms from previous genome-wide association studies. The polygenic risk score (PRS) for UC was obtained from the official UK Biobank PRS Release (15). In brief, the PRS was constructed by calculating the weighted sum of risk allele counts, where the weights were determined based on effect sizes derived from large-scale external genome-wide association studies. To account for population stratification, the raw genetic risk scores were subsequently centered and scaled based on genetically inferred ancestry, using the first 4 genetic principal components. This standardized methodology ensures the validity of the PRS across participants with diverse ancestral backgrounds.

### Ascertainment of outcomes and stratification factors

The primary outcome was incident UC, identified through linkage to hospital inpatient records, primary care data, and self-reported information using *International Classification of Diseases* (*ICD-10*) codes (K51). Comorbidities were also collected using *ICD-10* according to Table S1 (http://links.lww.com/CTG/B541) (16), including hypertension, diabetes, cardiovascular disease, and cancer. HDL and LDL were obtained using baseline blood sample tests (17). Follow-up began at the date of baseline assessment and continued until the date of diagnosis, death, or end of follow-up (October 31, 2022, for England; August 31, 2022, for Scotland; March 31, 2022 for Wales), whichever occurred first.

### Statistical analysis

Baseline characteristics were summarized across phospholipid quartiles using appropriate descriptive statistics. Continuous variables were presented as means with SDs and categorical variables as frequencies with percentages. Differences across quartiles were tested using analysis of variance for continuous variables and χ^2^ tests for categorical variables. The association between phospholipid levels and incident UC was assessed using Cox proportional hazards models with time since baseline as the time scale. We developed 3 sequential models: Model 1 was unadjusted; Model 2 adjusted for demographic, socioeconomic, and lifestyle factors; and Model 3 additionally adjusted for genetic risk score. The proportional hazards assumption was verified using Schoenfeld residuals. We also conducted generalized additive models to examine the potential nonlinear relationship between continuous phospholipid levels and UC risk. Subgroup analyses were performed by stratifying by age, sex, ethnicity, deprivation index, body mass index, smoking status, alcohol use, physical activity, healthy eating index, genetic risk score, and history of various comorbidities. Interaction terms were included to test for effect modification by these factors. In addition, we examined the associations between phospholipid concentrations within specific lipoprotein subfractions and UC risk using similar modeling approaches. All analyses were conducted using R version 4.2.0, with statistical significance set at *P* < 0.05 (2-tailed). Multiple imputation with chained equations was used to handle missing covariate data (<5% for all variables).

## RESULTS

### Participant characteristics and stratification by phospholipid levels

The analysis included 254,921 participants from the UK Biobank. Participants were stratified into quartiles (Q) based on their plasma total phospholipids in lipoprotein particles concentration: Q1 (lowest: 0.597 to <2.62 mmol/L, n = 63,721), Q2 (2.62 to <2.92 mmol/L, n = 63,722), Q3 (2.92 to <3.24 mmol/L, n = 63,747), and Q4 (highest: 3.24–7.23 mmol/L, n = 63,731). Baseline characteristics of the overall cohort and by phospholipid quartile are presented in Table 1.

**Table 1.** Baseline characteristics of participants in this study

Characteristic	Total phospholipids in lipoprotein particles					*P* value
	Overall N = 254,921	Q4 [3.24, 7.23] N = 63,731	Q3 [2.92, 3.24] N = 63,747	Q2 [2.62, 2.92] N = 63,722	Q1 [0.597, 2.62] N = 63,721	
Total phospholipids in lipoprotein particles						<0.001^a^
Mean ± SD	2.94 ± 0.48	3.56 ± 0.29	3.07 ± 0.09	2.77 ± 0.09	2.36 ± 0.21	
Median (Q1, Q3)	2.92 (2.62, 3.24)	3.48 (3.35, 3.69)	3.07 (2.99, 3.15)	2.78 (2.70, 2.85)	2.41 (2.24, 2.52)	
Min, max	0.60, 7.23	3.24, 7.23	2.92, 3.24	2.62, 2.92	0.60, 2.62	
Age, yr						<0.001^a^
Mean ± SD	57 ± 8	58 ± 7	57 ± 8	56 ± 8	56 ± 9	
Median (Q1, Q3)	58 (50, 63)	59 (53, 63)	58 (51, 63)	57 (49, 63)	57 (48, 64)	
Min, max	37, 73	37, 71	38, 71	39, 70	39, 73	
Age, yr, n (%)						<0.001^b^
<60	143,359 (56.2%)	33,264 (52.2%)	36,060 (56.6%)	38,015 (59.7%)	36,020 (56.5%)	
≥60	111,562 (43.8%)	30,467 (47.8%)	27,687 (43.4%)	25,707 (40.3%)	27,701 (43.5%)	
Sex, n (%)						<0.001^b^
Female	135,481 (53.1%)	45,493 (71.4%)	37,618 (59.0%)	30,924 (48.5%)	21,446 (33.7%)	
Male	119,440 (46.9%)	18,238 (28.6%)	26,129 (41.0%)	32,798 (51.5%)	42,275 (66.3%)	
Ethnics, n (%)						<0.001^b^
White	227,689 (89.3%)	58,301 (91.5%)	57,475 (90.2%)	56,748 (89.1%)	55,165 (86.6%)	
Other	27,232 (10.7%)	5,430 (8.5%)	6,272 (9.8%)	6,974 (10.9%)	8,556 (13.4%)	
Townsend Deprivation Index						<0.001^a^
Mean ± SD	−1.36 ± 3.07	−1.53 ± 2.95	−1.48 ± 2.99	−1.36 ± 3.07	−1.08 ± 3.23	
Median (Q1, Q3)	−2.21 (−3.68, 0.43)	−2.33 (−3.73, 0.13)	−2.30 (−3.72, 0.21)	−2.21 (−3.68, 0.46)	−1.97 (−3.56, 0.97)	
Min, max	−6.26, 10.88	−6.26, 10.82	−6.26, 10.42	−6.26, 10.56	−6.26, 10.88	
Townsend Deprivation Index, n (%)						<0.001^b^
Q4 [0.43, 10.9]	63,827 (25.0%)	14,636 (23.0%)	15,014 (23.6%)	16,050 (25.2%)	18,127 (28.4%)	
Q3 [−2.21, 0.43]	63,830 (25.0%)	16,089 (25.2%)	16,071 (25.2%)	15,883 (24.9%)	15,787 (24.8%)	
Q2 [−3.68, −2.21]	63,902 (25.1%)	16,499 (25.9%)	16,283 (25.5%)	15,887 (24.9%)	15,233 (23.9%)	
Q1 [−6.26, −3.68]	63,362 (24.9%)	16,507 (25.9%)	16,379 (25.7%)	15,902 (25.0%)	14,574 (22.9%)	
Body Mass Index						<0.001^a^
Mean ± SD	27.6 ± 4.8	27.2 ± 4.5	27.3 ± 4.7	27.5 ± 4.8	28.2 ± 5.1	
Median (Q1, Q3)	26.9 (24.3, 30.0)	26.7 (24.1, 29.7)	26.7 (24.2, 29.7)	26.8 (24.2, 29.9)	27.4 (24.7, 30.7)	
Min, max	12.1, 67.4	12.7, 64.8	13.9, 66.2	13.3, 65.0	12.1, 67.4	
Body Mass Index, n (%)						<0.001^b^
<30	190,863 (74.9%)	49,114 (77.1%)	48,775 (76.5%)	47,994 (75.3%)	44,980 (70.6%)	
≥30	64,058 (25.1%)	14,617 (22.9%)	14,972 (23.5%)	15,728 (24.7%)	18,741 (29.4%)	
Smoker, n (%)						<0.001^b^
Never	101,762 (39.9%)	25,191 (39.5%)	25,827 (40.5%)	25,872 (40.6%)	24,872 (39.0%)	
Ever	153,159 (60.1%)	38,540 (60.5%)	37,920 (59.5%)	37,850 (59.4%)	38,849 (61.0%)	
Alcohol user, n (%)						<0.001^b^
Never	11,132 (4.4%)	2,208 (3.5%)	2,551 (4.0%)	2,860 (4.5%)	3,513 (5.5%)	
Ever	243,789 (95.6%)	61,523 (96.5%)	61,196 (96.0%)	60,862 (95.5%)	60,208 (94.5%)	
Physical activity, MET						<0.001^a^
Mean ± SD	2,456 ± 2,378	2,513 ± 2,369	2,492 ± 2,393	2,445 ± 2,388	2,375 ± 2,359	
Median (Q1, Q3)	1,786 (1,058, 2,879)	1,786 (1,160, 2,988)	1,786 (1,097, 2,933)	1,786 (1,040, 2,853)	1,786 (972, 2,757)	
Min, max	0, 19,278	0, 19,278	0, 19,278	0, 19,278	0, 19,278	
Physical activity, MET, n (%)						<0.001^b^
H [1.79e+03, 1.93e+04]	156,539 (61.4%)	40,500 (63.5%)	39,718 (62.3%)	38,654 (60.7%)	37,667 (59.1%)	
L [0, 1.79e+03)	98,382 (38.6%)	23,231 (36.5%)	24,029 (37.7%)	25,068 (39.3%)	26,054 (40.9%)	
Healthy Eating Index, no.						<0.001^a^
Mean ± SD	3.40 ± 1.10	3.39 ± 1.07	3.39 ± 1.09	3.39 ± 1.11	3.42 ± 1.14	
Median (Q1, Q3)	3.00 (3.00, 4.00)	3.00 (3.00, 4.00)	3.00 (3.00, 4.00)	3.00 (3.00, 4.00)	3.00 (3.00, 4.00)	
Min, max	0.00, 8.00	0.00, 8.00	0.00, 8.00	0.00, 8.00	0.00, 8.00	
Healthy Eating Index, no., n (%)						<0.001^b^
H [3, 8]	198,623 (77.9%)	50,148 (78.7%)	49,766 (78.1%)	49,451 (77.6%)	49,258 (77.3%)	
L [0, 3)	56,298 (22.1%)	13,583 (21.3%)	13,981 (21.9%)	14,271 (22.4%)	14,463 (22.7%)	
Ulcerative colitis genetic risk						0.002^a^
Mean ± SD	0.13 ± 0.99	0.11 ± 0.99	0.12 ± 0.99	0.13 ± 0.98	0.13 ± 0.99	
Median (Q1, Q3)	0.13 (−0.54, 0.79)	0.12 (−0.56, 0.78)	0.12 (−0.55, 0.79)	0.13 (−0.53, 0.79)	0.13 (−0.53, 0.80)	
Min, max	−4.56, 4.55	−4.56, 4.30	−4.04, 4.55	−4.16, 4.33	−3.88, 4.08	
Ulcerative colitis genetic risk, n (%)						0.004^b^
Q1 [−4.56, −0.542]	63,730 (25.0%)	16,251 (25.5%)	16,088 (25.2%)	15,619 (24.5%)	15,772 (24.8%)	
Q2 [−0.542, 0.126]	63,730 (25.0%)	15,802 (24.8%)	15,971 (25.1%)	16,055 (25.2%)	15,902 (25.0%)	
Q3 [0.126, 0.791]	63,730 (25.0%)	15,905 (25.0%)	15,848 (24.9%)	16,071 (25.2%)	15,906 (25.0%)	
Q4 [0.791, 4.55]	63,731 (25.0%)	15,773 (24.7%)	15,840 (24.8%)	15,977 (25.1%)	16,141 (25.3%)	
Cancer, n (%)						<0.001^b^
No	234,112 (91.8%)	57,592 (90.4%)	58,590 (91.9%)	58,807 (92.3%)	59,123 (92.8%)	
Yes	20,809 (8.2%)	6,139 (9.6%)	5,157 (8.1%)	4,915 (7.7%)	4,598 (7.2%)	
Diabetes, n (%)						<0.001^b^
No	241,885 (94.9%)	62,725 (98.4%)	62,086 (97.4%)	60,971 (95.7%)	56,103 (88.0%)	
Yes	13,036 (5.1%)	1,006 (1.6%)	1,661 (2.6%)	2,751 (4.3%)	7,618 (12.0%)	
Cardiovascular Disease, n (%)						<0.001^b^
No	244,801 (96.0%)	62,480 (98.0%)	62,117 (97.4%)	61,463 (96.5%)	58,741 (92.2%)	
Yes	10,120 (4.0%)	1,251 (2.0%)	1,630 (2.6%)	2,259 (3.5%)	4,980 (7.8%)	
Hypertension, n (%)						<0.001^b^
No	185,866 (72.9%)	48,751 (76.5%)	48,388 (75.9%)	47,077 (73.9%)	41,650 (65.4%)	
Yes	69,055 (27.1%)	14,980 (23.5%)	15,359 (24.1%)	16,645 (26.1%)	22,071 (34.6%)	
Incident ulcerative colitis, n (%)	1,330 (0.52%)	301 (0.47%)	302 (0.47%)	325 (0.51%)	402 (0.63%)	<0.001^b^

MET, metabolic equivalent of task.

a One-way analysis of means.

b Pearson χ^2^ test.

Significant differences were observed across quartiles for all baseline characteristics (*P* < 0.001 for all comparisons unless otherwise specified). Participants in the highest phospholipid quartile (Q4) were older (mean age 58 ± 7 years vs 56 ± 9 years in Q1), more likely to be female (71.4% vs 33.7% in Q1), and predominantly of White ethnicity (91.5% vs 86.6% in Q1). Regarding baseline comorbidities, participants in Q4 exhibited a significantly lower prevalence of cardiovascular disease (2.0% vs 7.8%), diabetes (1.6% vs 12.0%), and hypertension (23.5% vs 34.6%) compared with those in Q1 (all *P* < 0.001), whereas the prevalence of cancer was higher (9.6% vs 7.2%). During the follow-up period, the incidence of UC decreased progressively across higher quartiles, with 0.47% in Q4 compared with 0.63% in Q1. Conversely, they exhibited a lower mean body mass index (27.2 ± 4.5 kg/m^2^ vs 28.2 ± 5.1 kg/m^2^ in Q1), lower Townsend Deprivation Index scores (indicating less deprivation; mean −1.53 ± 2.95 vs −1.08 ± 3.23 in Q1), and were more likely to be ever alcohol users (96.5% vs 94.5% in Q1). Physical activity levels (MET-min/week) were slightly higher in Q4 compared with Q1 (mean 2,513 ± 2,369 vs 2,375 ± 2,359), whereas the Healthy Eating Index showed minimal variation across groups. The distribution of UC genetic risk scores also differed significantly across quartiles (*P* = 0.002 for continuous, *P* = 0.004 for quartiles of genetic risk).

### Association of phospholipids with incident UC

**Figure 2.**
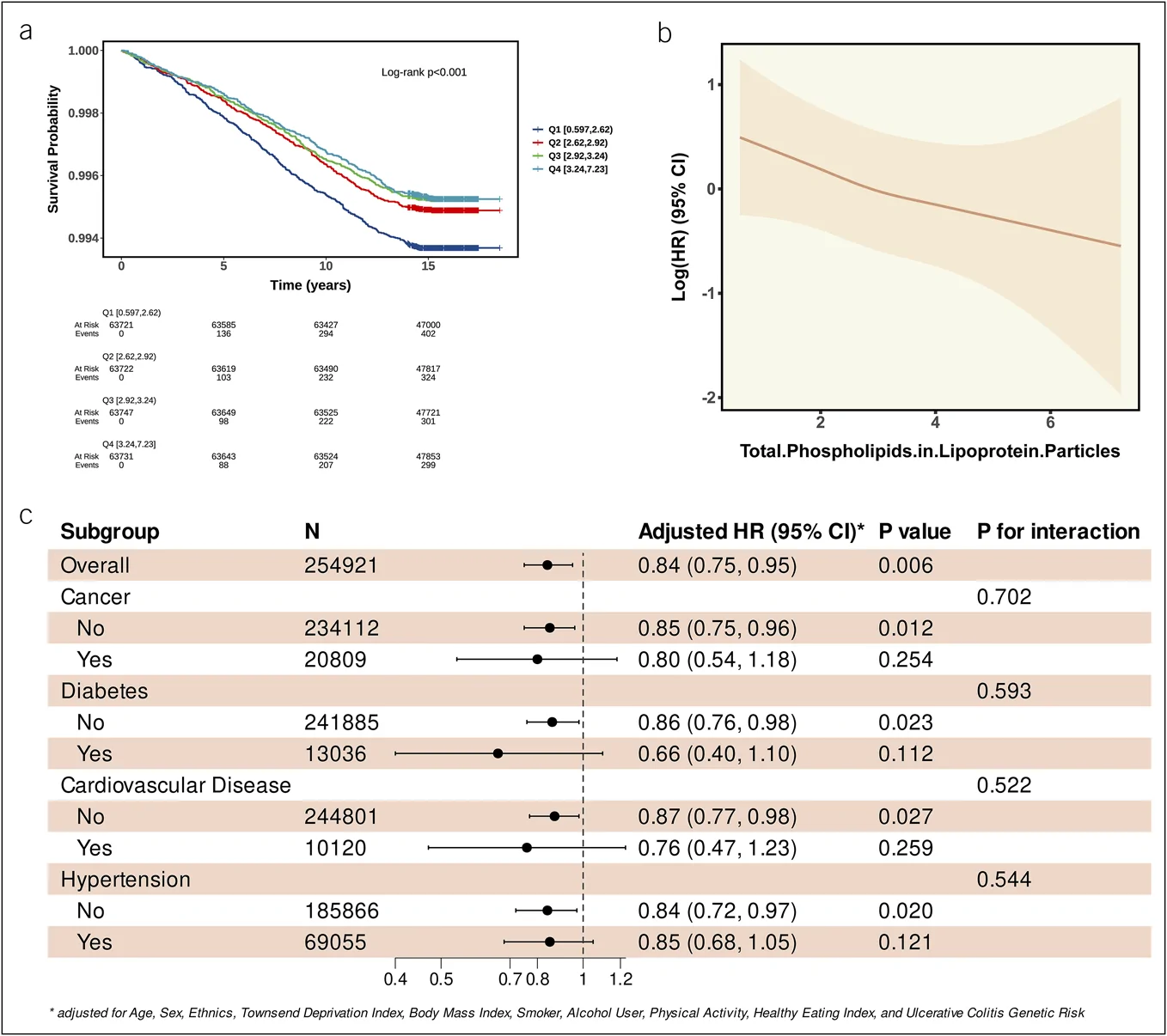
Association between plasma total phospholipids in lipoprotein particles and ulcerative colitis risk. (**a**) Kaplan-Meier survival curves showing cumulative incidence of ulcerative colitis across quartiles of phospholipid levels over follow-up time in 15 years, demonstrating significantly different survival probabilities (log rank *P* < 0.001). (**b**) Generalized additive model plot illustrating the continuous dose-response relationship between phospholipid levels and log hazard ratio for ulcerative colitis, with shaded area representing 95% CI. (**c**) Subgroup analysis table presenting adjusted hazard ratios across clinical subgroups including cancer, diabetes, cardiovascular disease, and hypertension status, showing sample sizes, point estimates with CIs, and interaction *P* values. CI, confidence interval; HR, hazard ratio.

During follow-up, 1,330 incident cases of UC were identified. Table 2 and Figure 2a presents the hazard ratios (HRs) for the association between plasma total phospholipids in lipoprotein particles and incident UC.

**Table 2.** Association of plasma total phospholipids in lipoprotein particles with the incidence of ulcerative colitis

Characteristic	Model 1					Model 2					Model 3				
	N	Event N	HR	95% CI	*P* value	N	Event N	HR	95% CI	*P* value	N	Event N	HR	95% CI	*P* value
Total phospholipids in lipoprotein particles (continuous)	254,921	1,330	0.77	0.69, 0.87	<0.001	254,921	1,330	0.84	0.75, 0.95	0.005	254,921	1,330	0.84	0.75, 0.95	0.006
Total phospholipids in lipoprotein particles															
Q1 [0.597, 2.62]	63,721	402	—	—		63,721	402	—	—		63,721	402	—	—	
Q2 [2.62, 2.92]	63,722	325	0.81	0.70, 0.93	0.004	63,722	325	0.86	0.74, 1.00	0.048	63,722	325	0.86	0.74, 1.00	0.048
Q3 [2.92, 3.24]	63,747	302	0.75	0.65, 0.87	<0.001	63,747	302	0.82	0.70, 0.95	0.009	63,747	302	0.82	0.70, 0.95	0.010
Q4 [3.24, 7.23]	63,731	301	0.75	0.64, 0.87	<0.001	63,731	301	0.83	0.71, 0.97	0.017	63,731	301	0.83	0.71, 0.97	0.020
*P* for trend					<0.001					0.011					0.013

Model 1: no covariates were adjusted. Model 2: adjusted for age, sex, ethnics, Townsend Deprivation Index, body mass index, smoker, alcohol user, physical activity, and Healthy Eating Index. Model 3: adjusted for age, sex, ethnics, Townsend Deprivation Index, body mass index, smoker, alcohol user, physical activity, Healthy Eating Index, and ulcerative colitis genetic risk.

CI, confidence interval; HR, hazard ratio.

In the unadjusted model (Model 1) and Figure 2a, both continuous phospholipid levels and quartile membership showed a significant inverse association with UC risk (Log Rank: *P* < 0.001). Each unit increase in phospholipids was associated with a 23% lower risk of UC (HR 0.77, 95% confidence interval [CI]: 0.69–0.87; *P* < 0.001). Compared with the lowest quartile (Q1), participants in higher quartiles had significantly lower risks, with the protective effect appearing to plateau at the third quartile: Q2 (HR 0.81, 95% CI 0.70–0.93; *P* = 0.004), Q3 (HR 0.75, 95% CI 0.65–0.87; *P* < 0.001), and Q4 (HR 0.75, 95% CI 0.64–0.87; *P* < 0.001), while maintaining a significant overall trend (*P* for trend < 0.001).

After adjustment for demographics (age, sex, ethnicity), socioeconomic status (Townsend Deprivation Index), and lifestyle factors (body mass index, smoking status, alcohol use, physical activity, healthy eating index) in Model 2, the associations attenuated but remained statistically significant. Each unit increase in phospholipids was associated with a 16% lower UC risk (HR 0.84, 95% CI 0.75–0.95; *P* = 0.005). The quartile analysis showed HRs of 0.86 (95% CI 0.74–1.00; *P* = 0.048) for Q2, 0.82 (95% CI 0.70–0.95; *P* = 0.009) for Q3, and 0.83 (95% CI 0.71–0.97; *P* = 0.017) for Q4 compared with Q1. The trend remained significant (*P* for trend = 0.011). Further adjustment for UC genetic risk score in Model 3 did not materially change the results. The continuous phospholipid association HR was 0.84 (95% CI 0.75–0.95; *P* = 0.006). Quartile HRs were Q2: 0.86 (95% CI 0.74–1.00; *P* = 0.048), Q3: 0.82 (95% CI 0.70–0.95; *P* = 0.010), Q4: 0.83 (95% CI 0.71–0.97; *P* = 0.020), and the test for trend remained significant (*P* for trend = 0.013). Consistent with the unadjusted model, the risk reduction in Models 2 and 3 was most pronounced up to Q3, after which the HRs stabilized, suggesting a potential threshold effect.

Figure 2b illustrates the continuous dose-response relationship between plasma total phospholipids in lipoprotein particles and the log-transformed HR for the study outcome. The generalized additive model demonstrates a clear, approximately linear inverse association across the clinically observed range of phospholipid levels (approximately 1–6 mmol/L). The central trend line, representing the point estimates of log(HR), shows a consistent negative slope throughout the exposure range, indicating that higher concentrations of phospholipids are associated with progressively lower log(HR) values (Tables S2–S4, http://links.lww.com/CTG/B541). However, in alignment with the quartile analysis, the curve suggests that the risk reduction may reach a plateau at higher phospholipid concentrations. This translates to a protective effect where increasing phospholipid levels correspond to a decreasing HR.

### Subgroup analysis of the association

**Figure 3.**
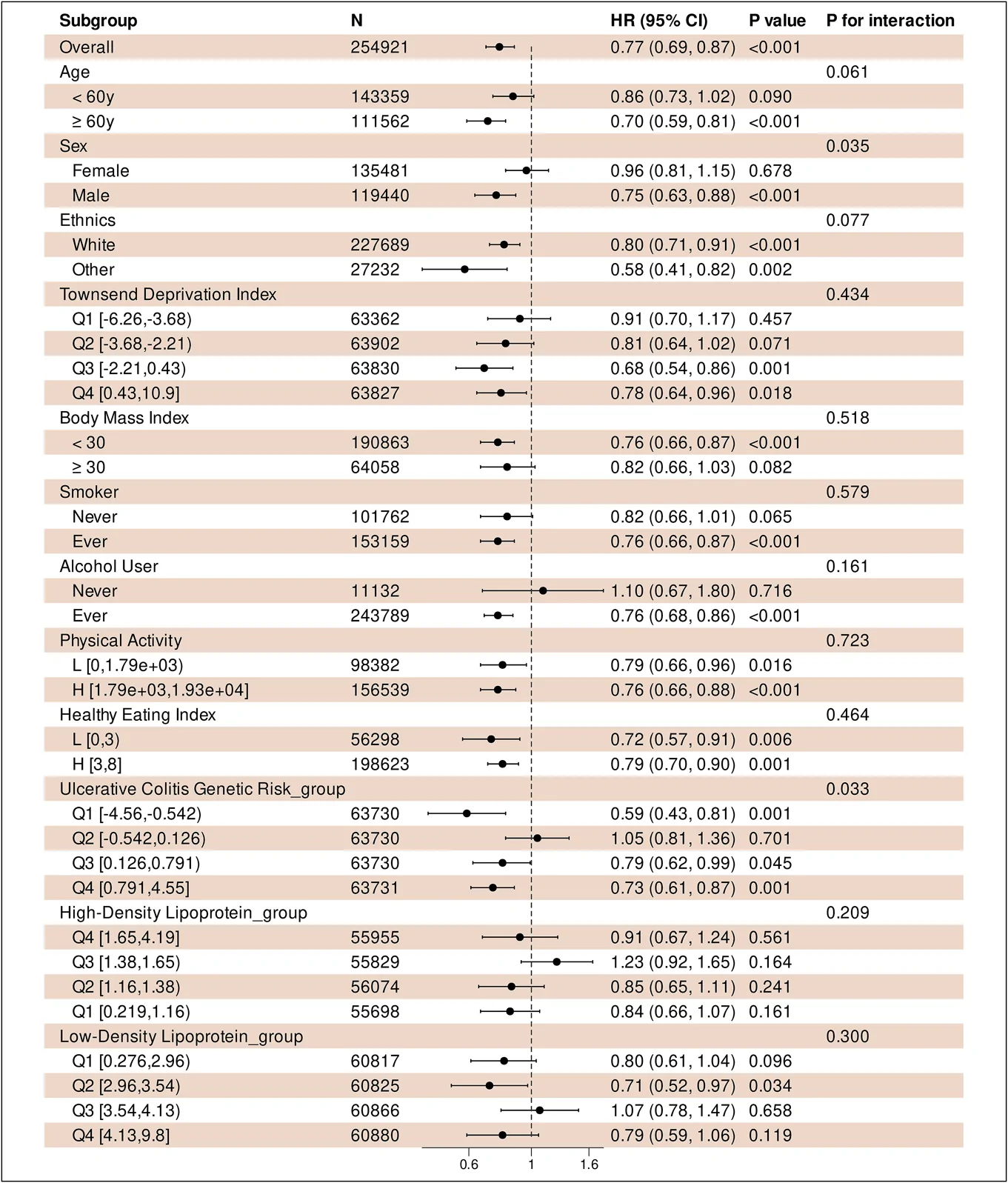
Stratified analysis of hazard ratios for ulcerative colitis across demographic, lifestyle, and genetic subgroups. The comprehensive table displays subgroup-specific associations including age, sex, ethnicity, socioeconomic status (Townsend Deprivation Index quartiles), body mass index, smoking status, alcohol use, physical activity levels, healthy eating index, genetic risk quartiles for ulcerative colitis, and lipoprotein subgroups. Each row presents subgroup sample size, unadjusted hazard ratio with 95% CI, *P* value for the association, and *P* value for interaction testing where applicable. CI, confidence interval; HR, hazard ratio.

The results of the subgroup analysis are detailed in the forest plot presented in Figures 2c and 3. The protective effect seemed generally consistent across most predefined patient subgroups, as indicated by the nonsignificant *P* values for interaction. Specifically, the inverse association remained significant among participants without a history of cancer (HR 0.85, 95% CI: 0.75–0.96; *P* = 0.012) and without a history of cardiovascular disease (HR 0.87, 95% CI: 0.77–0.98; *P* = 0.027). Although point estimates suggested a potentially stronger protective effect in subgroups with diabetes (HR 0.66) or cardiovascular disease (HR 0.76), the CIs for these subgroups were wider and crossed unity, likely due to smaller sample sizes, resulting in nonsignificant *P* values. Similarly, the association was significant in subgroups without hypertension (HR 0.84, 95% CI: 0.72–0.97; *P* = 0.020) and showed a consistent, though nonsignificant, trend among those with hypertension. Moreover, the beneficial effect seemed particularly pronounced in older participants (≥60 years: HR 0.70, 95% CI: 0.59–0.81, *P* < 0.001) compared with those aged younger than 60 years (HR 0.86, 95% CI: 0.73–1.02, *P* = 0.090), with a borderline significant interaction *P* value (0.061). A statistically significant interaction was observed by sex (*P* = 0.035), with men demonstrating a stronger protective effect (HR 0.75, 95% CI: 0.63–0.88, *P* < 0.001) than women (HR 0.96, 95% CI: 0.81–1.15, *P* = 0.678). Ethnicity subgroup analysis showed significant protection in both White participants (HR 0.80, 95% CI: 0.71–0.91, *P* < 0.001) and those of other ethnicities (HR 0.58, 95% CI: 0.41–0.82, *P* = 0.002), though the point estimate suggested possibly stronger protection in non-White groups (*P* for interaction = 0.077). The association remained significant across all Townsend deprivation quartiles, with particularly strong effects in Q3 (HR 0.68, 95% CI: 0.54–0.86, *P* = 0.001), without significant interaction (*P* = 0.434). Similarly consistent effects were observed across body mass index categories, smoking status, alcohol use, physical activity levels, and healthy eating index categories without significant interactions. Most notably, a significant interaction was observed across genetic risk quartiles for UC (*P* = 0.033), with the strongest protective effect evident in the lowest genetic risk quartile (Q1: HR 0.59, 95% CI: 0.43–0.81, *P* = 0.001). No significant interactions were observed with HDL or LDL subgroup classifications. The generally consistent direction of effect across most subgroups, despite variation in magnitude, strengthens the robustness of the primary finding.

### Association across lipoprotein subfractions

**Figure 4.**
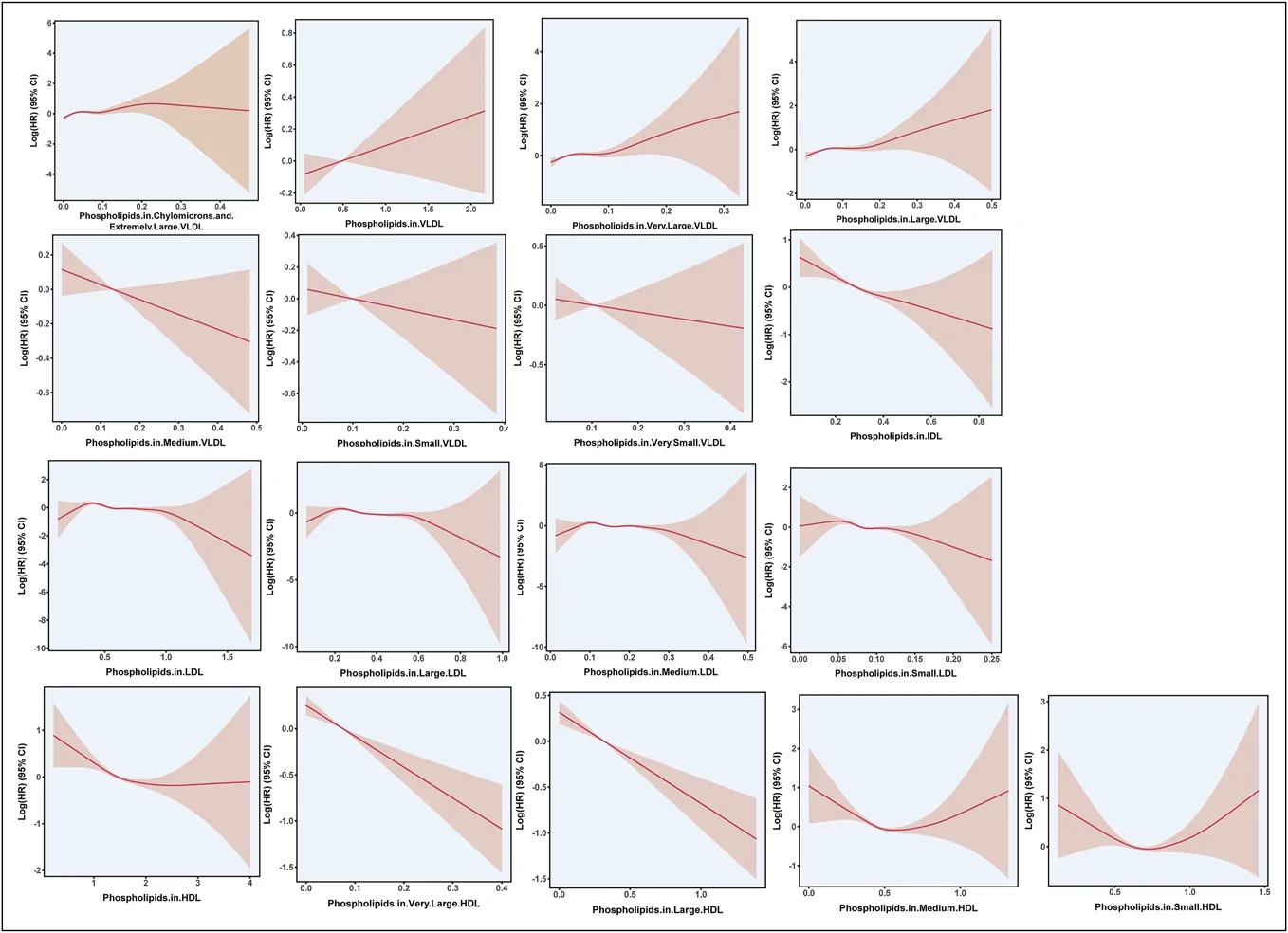
Dose-response associations between absolute phospholipid concentrations in lipoprotein subfractions and the risk of incident ulcerative colitis. The solid red lines represent the fitted log hazard ratios [log(HR)] derived from generalized additive models evaluating absolute phospholipid concentrations across lipoprotein subfractions. The shaded areas indicate the 95% CIs. The plots are organized by lipoprotein class and particle size. The association patterns were heterogeneous across subfractions, with inverse patterns mainly observed for HDL-, IDL-, and LDL-related phospholipids, whereas chylomicron- and several VLDL-related phospholipids showed null or positive/nonprotective patterns. CI, confidence interval; HDL, high-density lipoprotein; HR, hazard ratio; IDL, intermediate-density lipoprotein; LDL, low-density lipoprotein; VLDL, very low-density lipoprotein.

Figure 4 provides a comprehensive visualization of the dose-response associations between absolute phospholipid concentrations within specific lipoprotein subfractions and incident UC. The fitted generalized additive models revealed considerable heterogeneity across lipid components and particle sizes. In the fully adjusted Cox models, inverse associations were mainly observed for phospholipids carried by HDL, intermediate-density lipoprotein [IDL], and LDL particles and several related subfractions, including large and medium HDL as well as large, medium, and small LDL particles (Table S5, http://links.lww.com/CTG/B541). Evidence was less consistent for very large HDL and small HDL, where inverse associations were limited to selected higher quartiles.

By contrast, phospholipids in chylomicrons and extremely large VLDL, very large VLDL, and large VLDL did not demonstrate inverse associations with UC risk. The corresponding dose-response curves were largely flat or upward-sloping, and the quartile-based estimates suggested positive or nonprotective associations rather than risk reduction. Phospholipids in medium, small, and very small VLDL were generally close to null. These findings clarify that the inverse association observed for total phospholipids was not uniformly shared across all lipoprotein subfractions and should not be attributed to triglyceride-rich chylomicron/VLDL-related particles. Furthermore, we evaluated the relative percentage of phospholipids to total lipids within these subfractions (Figure S1, http://links.lww.com/CTG/B541). In contrast to the findings for absolute concentrations, the relative percentage measures did not show a consistent inverse pattern across lipoprotein subclasses. In fact, most of the subfractions showed either a null or a positive association when evaluated as a relative proportion. This disparity suggests that the absolute quantity of protective phospholipids circulating in specific particles, rather than their relative compositional percentage within the lipid droplet, is the primary driver of the observed risk reduction. To understand the relationship between phospholipids and the overall fat content of lipoproteins, we assessed their correlation with the primary core lipids. As biologically anticipated, the absolute concentration of total phospholipids demonstrated a strong positive correlation with total cholesterol (Pearson *r* = 0.79, *P* < 0.001) and a significant positive correlation with triglycerides (Pearson *r* = 0.24, *P* < 0.001) (Table S8 and Figure S2, http://links.lww.com/CTG/B541). However, as demonstrated in our subfraction percentage analysis (Figure S1, http://links.lww.com/CTG/B541), the relative proportion of phospholipids to total lipids did not show a consistent protective trend. This contrast suggests that the protective association is driven by the absolute circulating mass of phospholipids rather than merely reflecting a higher overall lipid content or a specific proportional composition.

## DISCUSSION

### Summary of findings

This large prospective cohort study of 254,921 participants from the UK Biobank provides evidence for an inverse association between plasma levels of total phospholipids in lipoprotein particles and the risk of incident UC. Our analysis demonstrated a nonlinear dose-response pattern, with participants in the highest phospholipid quartile exhibiting a 17% lower risk of developing UC compared with those in the lowest quartile, even after comprehensive adjustment for demographic, socioeconomic, lifestyle, and genetic risk factors. However, the risk reduction was not strictly monotonic. The quartile estimates reached their lowest point around the third quartile and were similar in the fourth quartile, supporting a plateau/threshold pattern at higher phospholipid concentrations rather than a continuous linear decrease in risk. Notably, this relationship remained generally consistent across most population subgroups defined by age, comorbidity status, and lifestyle factors. Furthermore, our extensive analysis of lipoprotein subfractions revealed substantial heterogeneity across particle classes and sizes. The inverse associations were mainly observed for phospholipids carried by HDL, IDL, and LDL particles and several related subfractions, whereas chylomicron- and several VLDL-related phospholipids did not show inverse associations and in some cases showed positive or nonprotective patterns. Therefore, the association observed for total phospholipids should be interpreted as an aggregate metabolic signal rather than evidence that all phospholipid-carrying lipoprotein particles are protective.

### Comparison with previous literature

Our findings contribute novel insights to the evolving understanding of the role of lipids and metabolism in inflammatory bowel disease pathogenesis. However, previous studies have predominantly focused on circulating cholesterol levels (18) with mixed results in animals (19,20). Our study is among the first to comprehensively examine phospholipids—a distinct and functionally important class of lipids—in relation with UC risk. The observed inverse association aligns with emerging evidence suggesting a protective role for certain lipid components in intestinal inflammation. For instance, epidemiological studies have reported reduced risks of Crohn's disease associated with altered phospholipids (21), though evidence for UC has been less consistent. Our findings extend this literature by demonstrating that phospholipids, which represent a more direct measure of lipoprotein particle metabolism and function, exhibit a robust protective association.

The subfraction-specific findings require a more nuanced interpretation than a uniform protective effect across all lipoprotein classes. While HDL particles have received the most attention for their putative anti-inflammatory properties, our results suggest that inverse associations were also present for phospholipids carried by IDL and LDL particles and several related subfractions. However, this pattern was not observed for all apoB-containing particles (22,23). In particular, chylomicron- and large VLDL-related phospholipids did not demonstrate inverse associations with UC risk, and some estimates were positive or nonprotective. These findings suggest that the biological context of the lipoprotein carrier, particle size, and triglyceride-rich vs cholesterol-rich particle metabolism may modify the association between phospholipids and UC risk. Therefore, our results should not be interpreted as showing that phospholipids in all lipoprotein particles are protective, but rather that total phospholipid levels may capture an aggregate metabolic phenotype in which HDL-, IDL-, and LDL-related phospholipids seem to contribute more strongly to the inverse association. The finding that the association was independent of genetic predisposition to UC, as measured by polygenic risk scores, further supports phospholipids as an independent metabolic biomarker, although causal and therapeutic implications require further investigation.

Our subgroup analysis revealed a significant sex difference (*P*_interaction_ = 0.035), where the protective association of phospholipids against incident UC was pronounced in men but nonsignificant in women. This discrepancy may be largely explained by baseline physiological variations and a threshold effect. In our cohort, women exhibited significantly higher baseline plasma phospholipid levels than men (mean 3.07 vs 2.80 mmol/L, *P* < 0.001), a phenomenon likely driven by the regulatory effects of estrogen on lipid synthesis. Consequently, the average female participant was already situated within the concentration range corresponding to the third quartile (2.92–3.24 mmol/L) (Table S7, http://links.lww.com/CTG/B541). As we observed that the protective benefits plateau at these higher concentrations, the marginal risk reduction of further phospholipid elevation in women is understandably attenuated. Furthermore, subsequent age-stratification among women indicated that in the older demographic (>50 years, a proxy for postmenopause), the HR trended toward a protective direction (HR 0.87, 95% CI 0.72–1.06), suggesting that hormonal changes may dynamically influence the interplay between phospholipid metabolism and UC pathogenesis (Table S6, http://links.lww.com/CTG/B541).

### Potential biological mechanisms

Several biological mechanisms could explain the observed protective association between phospholipids and UC risk. Phospholipids are fundamental components of cellular membranes and are essential for maintaining intestinal barrier integrity (8). Higher systemic phospholipids levels may reflect enhanced capacity for mucosal repair and maintenance of the epithelial barrier, which is fundamentally disrupted in UC (24). Second, phospholipids serve as precursors for anti-inflammatory and pro-resolving lipid mediators (25), such as platelet-activating factor and lysophosphatidic acid, which play crucial roles in modulating immune responses and resolving inflammation (26). In addition, phospholipids in lipoprotein particles, particularly HDL, may contribute to neutralizing bacterial lipopolysaccharides and other microbial products that can trigger intestinal inflammation (27). The heterogeneity across lipoprotein subclasses argues against a simple interpretation that all phospholipid molecules or all lipoprotein carriers are uniformly protective. The inverse associations observed for HDL-, IDL-, and LDL-related phospholipids may reflect mechanisms related to epithelial membrane maintenance, anti-inflammatory lipid mediator pathways, and lipoprotein-mediated neutralization of microbial products. By contrast, the null or positive/nonprotective patterns observed for chylomicron- and VLDL-related phospholipids may reflect the distinct biology of triglyceride-rich particles, remnant metabolism, or residual metabolic confounding. These mechanistic explanations remain hypothesis-generating and require validation in experimental and independent prospective studies.

### Strengths and limitations

The major strengths of this study include its prospective design, large sample size, long follow-up period, and comprehensive assessment of covariates. The use of nuclear magnetic resonance spectroscopy allowed for detailed measurement of phospholipids across multiple lipoprotein subfractions, providing unprecedented resolution into lipid metabolism. The availability of genetic data enabled adjustment for genetic predisposition to UC, strengthening the robustness of our findings. However, several limitations should be acknowledged. Despite extensive adjustment for known confounders, residual confounding from unmeasured or imperfectly measured factors remains possible. Although phospholipids were measured before UC diagnosis, reverse causality cannot be entirely excluded, as subclinical inflammation might affect lipid metabolism. The UK Biobank participants are predominantly of White ethnicity and generally healthier than the general population, which may limit the generalizability of our findings. Finally, while we observed consistent associations across subgroups, some subgroup analyses were limited by reduced statistical power.

In conclusion, this large prospective study provides robust evidence that higher plasma levels of phospholipids in lipoprotein particles are associated with a reduced risk of incident UC. From a clinical perspective, phospholipids may represent a novel biomarker for identifying individuals at elevated risk for developing UC, potentially enabling earlier interventions. From a mechanistic standpoint, our results suggest that pathways involving phospholipid metabolism warrant further investigation as potential therapeutic targets for UC prevention or treatment. Future research should aim to validate these findings in independent cohorts, explore the specific molecular species of phospholipids that may be driving this association, and investigate the potential therapeutic utility of phospholipid-based interventions in experimental models of intestinal inflammation.

## CONFLICTS OF INTEREST

**Guarantor of the article:** Shaojun Liu, MD.

**Specific author contributions:** All authors read and approved the final manuscript. S.L. conceived and designed the project. J.T. analyzed the data. D.Z. drafted the manuscript. All authors read and approved the final manuscript.

**Financial support:** This article was supported by the Natural Science Foundation of Hunan Province by SJ.L (No. 2020JJ8107). The study sponsors have no interference on the study design, collection, analysis, and interpretation of the data and in the writing of the report.

**Potential competing interests:** The authors declare that there is no conflict of interests in this study.

**Ethical statement:** This research has been approved by UK biobank (No. 104784).

**IRB approval statement:** This study complied with the ethics approval and consent of the UK biobank and was approved by the UK biobank (No. 104784).

**Study Highlights**

**WHAT IS KNOWN**

✓ Ulcerative colitis (UC) pathogenesis involves complex genetic and environmental interactions.
✓ Previous research suggests that metabolic factors may influence inflammatory bowel disease risk.
✓ Identifying modifiable risk factors of UC prevention remains a significant challenge.

**WHAT IS NEW HERE**

✓ Higher plasma phospholipid levels are inversely associated with ulcerative colitis risk.
✓ A linear dose-response relationship exists between phospholipids and reduced UC incidence.
✓ Phospholipid metabolism may offer novel biomarkers for UC risk stratification.
✓ This study identifies potential therapeutic targets for ulcerative colitis prevention.

## Supplementary Material

## ABBREVIATIONS:

ApoB: apolipoprotein B
BMI: body mass index
CI: confidence interval
HDL: high-density lipoprotein
HR: hazard ratio
IBD: inflammatory bowel disease
ICD-10: International Classification of Diseases, 10th Revision
IDL: intermediate-density lipoprotein
LDL: low-density lipoprotein
MET: metabolic equivalent of task
NHS: National Health Service
NMR: nuclear magnetic resonance
PRS: polygenic risk score
UC: ulcerative colitis
UK Biobank: United Kingdom Biobank
VLDL: very low-density lipoprotein
