# Supplementary material for: Plasma Phospholipids and Incident Ulcerative Colitis: A Prospective Cohort Study in the UK Biobank
Source: Clin Transl Gastroenterol. 2026 Jun 4;17(8):e01051. doi: 10.14309/ctg.0000000000001051 (PMC13502777; doi:10.14309/ctg.0000000000001051)

SUPPLEMENTAL MATERIALS

Plasma Phospholipids and Incident Ulcerative Colitis: A Prospective Cohort Study in the UK Biobank

eTable 1. Sources and codes of outcome and covariates in UK Biobank.

Disease	ICD-9	ICD-10	Self-report	Other sources
**Main studied diseases**				
Ulcerative colitis	556X	K51	1463	Feild ID: 131626, 131627
**History of diseases**				
Hypertension	401X, 402X, 403X, 4043X, 405	I10X, I11X, I12X, I13X, I14X	1065, 1072, 1073	Feild ID: 6150 = 4; 6177 = 2;<br>6153=2;<br>4079>90mmHg;<br>4080 >140mmHg
Diabetes	250X	E10X, E11X, E12X, E13X,<br>E14X	1220, 1221, 1222,1223	Feild ID: 2443 = 1; 6177 = 3;<br>6153=3;<br>30750 >47mmol/mol
Cardiovascular disease	391X, 393X, 394X, 395X, 396X,<br>397X, 398X, 410X, 411X, 412X,<br>413X, 414X, 415X, 416X, 417X,<br>42X, 430X, 431X, 434X, 436X	I01X, I05X, I06X, I07X, I09X, I13X, I2X, I3X, I4X, I5X, I60X, I61X, I63X, I64X	1074, 1075, 1076, 1077, 1078,<br>1079, 1080, 1081, 1086, 1426,<br>1471, 1483, 1484, 1485, 1486,<br>1487, 1488, 1489, 1490, 1491,<br>1583, 1584, 1585, 1586, 1587,<br>1588, 1589, 1590	Feild ID: 6150 = 1, 2, 3
Cancer	14X, 15X, 16X, 17X, 18X, 19X,<br>20X	C0X, C1X, C2X, C3X, C4X,<br>C5X, C6X, C7X, C8X, C9X	10X	

Abbreviations: International Classification of Diseases: 10th Revision (ICD-10) code and 9th Revision (ICD-9) code

eTable 2. Approximate significance of smooth terms in generalized additive models

Characteristics	Effective degree of freedom (edf)	Reference degrees of freedom (Ref.df)	Chi.sq	P value
s(Total.Phospholipids.in.Lipoprotein.Particles)	1.472354	1.828437	7.533604	0.013

s: Smooth Function

eTable 3. Parametric coefficients (Linear terms effect) in generalized additive models

Characteristics	Estimate	SE	z value	P value
Age	0.016401056527	0.00353015602	4.6459863	<0.001
SexMale	0.220725635596	0.05863481178	3.7644128	<0.001
EthnicsOther	0.027546392470	0.08934807605	0.3083043	0.758
Townsend.Deprivation.Index	0.039401552632	0.00867315198	4.5429335	<0.001
Body.Mass.Index	0.005633176504	0.00574280553	0.9809102	0.327
SmokerEver	0.436726056168	0.06180996641	7.0656252	<0.001
Alcohol.UserEver	-0.287275017057	0.12921200629	-2.2232842	0.026
Physical.Activity	0.000007502042	0.00001124422	0.6671913	0.505
Healthy.Eating.Index	-0.043583046673	0.02504297478	-1.7403303	0.082

SE: standard error

eTable 4. Goodness of fit measures in generalized additive models

df	logLik	AIC	BIC	deviance	df.residual	nobs	adj.r.squared	npar
10.47235	-16,472.73	32,967.12	33,023.35	16,472.58	254,910.5	1,330		18

AIC: Akaike information criterion, BIC: Bayesian information criterion

eTable 5. Association between specific phospholipids and the incidence of ulcerative colitis

Characteristic	N	Event N	HR	95% CI	p-value
Phospholipids in Chylomicrons and Extremely Large VLDL					
Q1 [1.48e-07,0.0128)	63,727	268	—	—	
Q2 [0.0128,0.0286)	63,730	320	1.19	1.02, 1.40	0.032
Q3 [0.0286,0.053)	63,733	370	1.38	1.18, 1.62	<0.001
Q4 [0.053,0.475]	63,731	372	1.39	1.19, 1.62	<0.001
Phospholipids in HDL					
Q1 [0.227,1.33)	63,700	411	—	—	
Q2 [1.33,1.52)	63,733	315	0.77	0.66, 0.89	<0.001
Q3 [1.52,1.74)	63,753	304	0.74	0.64, 0.86	<0.001
Q4 [1.74,4.01]	63,735	300	0.73	0.63, 0.85	<0.001
Phospholipids in IDL					
Q1 [0.0495,0.248)	63,728	402	—	—	
Q2 [0.248,0.29)	63,728	345	0.86	0.74, 0.99	0.036
Q3 [0.29,0.335)	63,733	303	0.75	0.65, 0.87	<0.001
Q4 [0.335,0.857]	63,732	280	0.7	0.60, 0.81	<0.001
Phospholipids in LDL					
Q1 [0.118,0.513)	63,725	412	—	—	
Q2 [0.513,0.604)	63,732	310	0.75	0.65, 0.87	<0.001
Q3 [0.604,0.702)	63,729	316	0.77	0.66, 0.89	<0.001
Q4 [0.702,1.7]	63,735	292	0.71	0.61, 0.82	<0.001
Phospholipids in Large HDL					
Q1 [5.19e-07,0.208)	63,724	390	—	—	
Q2 [0.208,0.288)	63,732	335	0.86	0.74, 0.99	0.04
Q3 [0.288,0.4)	63,733	334	0.86	0.74, 0.99	0.037
Q4 [0.4,1.39]	63,732	271	0.69	0.59, 0.81	<0.001
Phospholipids in Large LDL					
Q1 [0.0638,0.301)	63,723	411	—	—	
Q2 [0.301,0.355)	63,737	327	0.79	0.69, 0.92	0.002
Q3 [0.355,0.412)	63,729	311	0.76	0.65, 0.88	<0.001
Q4 [0.412,0.989]	63,732	281	0.68	0.59, 0.79	<0.001
Phospholipids in Large VLDL					
Q1 [5.74e-08,0.0404)	63,729	280	—	—	
Q2 [0.0404,0.0643)	63,731	345	1.23	1.05, 1.44	0.009
Q3 [0.0643,0.0936)	63,730	354	1.26	1.08, 1.48	0.003
Q4 [0.0936,0.499]	63,731	351	1.25	1.07, 1.47	0.005
Phospholipids in Medium HDL					
Q1 [3.26e-05,0.419)	63,728	406	—	—	
Q2 [0.419,0.482)	63,726	305	0.75	0.65, 0.87	<0.001
Q3 [0.482,0.55)	63,732	313	0.77	0.66, 0.89	<0.001
Q4 [0.55,1.32]	63,735	306	0.75	0.65, 0.87	<0.001
Phospholipids in Medium LDL					
Q1 [0.0134,0.134)	63,721	394	—	—	
Q2 [0.134,0.161)	63,716	316	0.8	0.69, 0.93	0.003
Q3 [0.161,0.191)	63,744	310	0.79	0.68, 0.91	0.001
Q4 [0.191,0.496]	63,740	310	0.79	0.68, 0.91	0.001
Phospholipids in Medium VLDL					
Q1 [2.52e-08,0.0976)	63,730	361	—	—	

Q2 [0.0976,0.128)	63,730	321	0.89	0.76, 1.03	0.124
Q3 [0.128,0.161)	63,730	324	0.9	0.77, 1.04	0.155
Q4 [0.161,0.482]	63,731	324	0.9	0.77, 1.04	0.153
Phospholipids in Small HDL					
Q1 [0.12,0.604)	63,728	364	—	—	
Q2 [0.604,0.662)	63,731	331	0.91	0.78, 1.05	0.209
Q3 [0.662,0.725)	63,723	301	0.83	0.71, 0.96	0.015
Q4 [0.725,1.46]	63,739	334	0.92	0.79, 1.06	0.253
Phospholipids in Small LDL					
Q1 [1.58e-07,0.0762)	63,730	401	—	—	
Q2 [0.0762,0.0881)	63,725	308	0.77	0.66, 0.89	<0.001
Q3 [0.0881,0.101)	63,721	316	0.79	0.68, 0.91	0.001
Q4 [0.101,0.25]	63,745	305	0.76	0.65, 0.88	<0.001
Phospholipids in Small VLDL					
Q1 [0.0105,0.0781)	63,730	344	—	—	
Q2 [0.0781,0.097)	63,728	329	0.96	0.82, 1.11	0.56
Q3 [0.097,0.118)	63,732	328	0.95	0.82, 1.11	0.533
Q4 [0.118,0.384]	63,731	329	0.96	0.82, 1.11	0.555
Phospholipids in VLDL					
Q1 [0.0437,0.353)	63,728	293	—	—	
Q2 [0.353,0.463)	63,730	374	1.28	1.10, 1.49	0.002
Q3 [0.463,0.593)	63,732	325	1.11	0.95, 1.30	0.199
Q4 [0.593,2.16]	63,731	338	1.15	0.99, 1.35	0.075
Phospholipids in Very Large HDL					
Q1 [6.46e-09,0.0463)	63,730	376	—	—	
Q2 [0.0463,0.0662)	63,728	356	0.95	0.82, 1.09	0.458
Q3 [0.0662,0.0957)	63,731	340	0.9	0.78, 1.05	0.177
Q4 [0.0957,0.4]	63,732	258	0.69	0.59, 0.80	<0.001
Phospholipids in Very Large VLDL					
Q1 [2.12e-07,0.0208)	63,726	282	—	—	
Q2 [0.0208,0.0358)	63,728	335	1.19	1.01, 1.39	0.033
Q3 [0.0358,0.0555)	63,735	355	1.26	1.08, 1.47	0.004
Q4 [0.0555,0.326]	63,732	358	1.27	1.09, 1.48	0.003
Phospholipids in Very Small VLDL					
Q1 [0.021,0.0876)	63,727	343	—	—	
Q2 [0.0876,0.103)	63,712	331	0.96	0.83, 1.12	0.643
Q3 [0.103,0.122)	63,731	325	0.95	0.81, 1.10	0.481
Q4 [0.122,0.428]	63,751	331	0.96	0.83, 1.12	0.634
Phospholipids to Total Lipids in Chylomicrons and Extremely Large VLDL percentage					
Q1 [0.000541,14.6)	63,708	268	—	—	
Q2 [14.6,15.8)	63,705	308	1.15	0.98, 1.35	0.095
Q3 [15.8,16.9)	63,756	371	1.38	1.18, 1.62	<0.001
Q4 [16.9,47.9]	63,752	383	1.43	1.22, 1.67	<0.001
Phospholipids to Total Lipids in IDL percentage					
Q1 [8.26,23.3)	63,681	283	—	—	
Q2 [23.3,23.9)	63,718	319	1.13	0.96, 1.32	0.144
Q3 [23.9,24.5)	63,777	335	1.18	1.01, 1.39	0.038
Q4 [24.5,40.8]	63,745	393	1.39	1.19, 1.62	<0.001
Phospholipids to Total Lipids in Large HDL percentage					

Q1 [0.00105,48.6)	63,686	260	—	—	
Q2 [48.6,50.1)	63,758	305	1.17	0.99, 1.38	0.06
Q3 [50.1,52.1)	63,740	370	1.42	1.21, 1.67	<0.001
Q4 [52.1,78.1]	63,737	395	1.52	1.30, 1.78	<0.001
Phospholipids to Total Lipids in Large LDL percentage					
Q1 [18.6,22)	63,641	331	—	—	
Q2 [22,22.5)	63,713	318	0.96	0.82, 1.12	0.6
Q3 [22.5,23)	63,815	318	0.96	0.82, 1.12	0.586
Q4 [23,51.2]	63,752	363	1.1	0.94, 1.27	0.231
Phospholipids to Total Lipids in Large VLDL percentage					
Q1 [0.000151,18.6)	63,722	263	—	—	
Q2 [18.6,19.9)	63,676	317	1.21	1.02, 1.42	0.024
Q3 [19.9,20.9)	63,790	367	1.39	1.19, 1.63	<0.001
Q4 [20.9,42.4]	63,733	383	1.46	1.25, 1.70	<0.001
Phospholipids to Total Lipids in Medium HDL percentage					
Q1 [0.0572,46.3)	63,672	250	—	—	
Q2 [46.3,47)	63,758	323	1.29	1.09, 1.52	0.002
Q3 [47,47.9)	63,740	344	1.37	1.17, 1.62	<0.001
Q4 [47.9,60.6]	63,751	413	1.65	1.41, 1.93	<0.001
Phospholipids to Total Lipids in Medium LDL percentage					
Q1 [21,25.6)	63,663	343	—	—	
Q2 [25.6,26.2)	63,763	353	1.03	0.89, 1.19	0.721
Q3 [26.2,26.7)	63,682	322	0.94	0.81, 1.09	0.411
Q4 [26.7,58]	63,813	312	0.91	0.78, 1.06	0.214
Phospholipids to Total Lipids in Medium VLDL percentage					
Q1 [4.81e-05,21.1)	63,703	389	—	—	
Q2 [21.1,22.3)	63,712	374	0.96	0.83, 1.11	0.584
Q3 [22.3,23.4)	63,741	295	0.76	0.65, 0.88	<0.001
Q4 [23.4,46.7]	63,765	272	0.7	0.60, 0.82	<0.001
Phospholipids to Total Lipids in Small HDL percentage					
Q1 [50.2,56.2)	63,693	321	—	—	
Q2 [56.2,57)	63,717	318	0.99	0.85, 1.16	0.903
Q3 [57,57.9)	63,740	356	1.11	0.95, 1.29	0.18
Q4 [57.9,65.1]	63,771	335	1.04	0.89, 1.22	0.593
Phospholipids to Total Lipids in Small LDL percentage					
Q1 [0.000475,30.1)	63,702	367	—	—	
Q2 [30.1,31.1)	63,722	305	0.83	0.71, 0.97	0.017
Q3 [31.1,32.2)	63,763	326	0.89	0.76, 1.03	0.116
Q4 [32.2,75.8]	63,734	332	0.9	0.78, 1.05	0.183
Phospholipids to Total Lipids in Small VLDL percentage					
Q1 [8.89,22.3)	63,714	434	—	—	
Q2 [22.3,23.6)	63,724	352	0.81	0.70, 0.93	0.003
Q3 [23.6,24.9)	63,749	286	0.66	0.57, 0.76	<0.001
Q4 [24.9,35.4]	63,734	258	0.59	0.51, 0.69	<0.001
Phospholipids to Total Lipids in Very Large VLDL percentage					
Q1 [0.00204,17.9)	63,696	312	—	—	
Q2 [17.9,18.8)	63,691	364	1.17	1.00, 1.36	0.045
Q3 [18.8,19.6)	63,758	338	1.08	0.93, 1.26	0.314
Q4 [19.6,34.7]	63,776	316	1.01	0.86, 1.18	0.887

Phospholipids to Total Lipids in Very Large HDL percentage					
Q1 [5.31e-05,41.8)	63,727	362	—	—	
Q2 [41.8,45.7)	63,727	330	0.91	0.79, 1.06	0.223
Q3 [45.7,48.8)	63,726	349	0.96	0.83, 1.12	0.626
Q4 [48.8,59.9]	63,741	289	0.8	0.68, 0.93	0.004
Phospholipids to Total Lipids in Very Small VLDL percentage					
Q1 [17.2,28.7)	63,679	279	—	—	
Q2 [28.7,29.2)	63,749	279	1	0.85, 1.18	0.993
Q3 [29.2,29.8)	63,712	364	1.31	1.12, 1.53	<0.001
Q4 [29.8,38.6]	63,781	408	1.46	1.26, 1.70	<0.001

Abbreviations: CI = Confidence Interval, HR = Hazard Ratio

Adjusted for Model 3 covariates: Age, Sex, Ethnicity, Townsend Deprivation Index, Body Mass Index, Smoking status, Alcohol use, Physical Activity, Healthy Eating Index, and Ulcerative Colitis Genetic Risk Score.

eTable 6. Association of plasma total phospholipids with incident UC stratified by female age

**Subgroup**	**N**	**Event N**	**HR (95% CI) ***	**p-value**
**Female Age Stratification**	135,481	601	0.96 (0.81, 1.15)	0.678
Age < 50 years	30,964	112	1.36 (0.90, 2.07)	0.144
Age ≥ 50 years	104,517	489	0.87 (0.72, 1.06)	0.167

* Adjusted for Model 3 covariates: Age, Sex, Ethnicity, Townsend Deprivation Index, Body Mass Index, Smoking status, Alcohol use, Physical Activity, Healthy Eating Index, and Ulcerative Colitis Genetic Risk Score.
Abbreviations: CI = Confidence Interval, HR = Hazard Ratio

eTable 7. Baseline plasma total phospholipids in lipoprotein particles stratified by sex

**Characteristic**	**Overall (N = 254,921)**	**Male (N = 119,440)**	**Female (N = 135,481)**	**p-value***
**Total Phospholipids in Lipoprotein Particles (mmol/L)**				
Mean ± SD	2.94 ± 0.48	2.80 ±0.45	3.07 ± 0.46	< 0.001

* p-value was calculated using the Welch Two Sample t-test for continuous variables comparing males and females.
Abbreviations: SD = Standard Deviation

eTable 8. Correlation between plasma total phospholipids in lipoprotein particles and primary lipid components

**Lipid Component**	**Pearson Correlation Coefficient (r)**	**95% CI**	**p-value**
**Total Cholesterol**	0.79	0.78, 0.79	< 0.001
**Triglycerides**	0.24	0.23, 0.24	< 0.001

* Correlations were calculated using Pearson's product-moment correlation to assess the linear relationship between absolute concentrations.
Abbreviations: CI = Confidence Interval

eFigure 1. Dose-response associations between the relative percentage of phospholipids to total lipids in lipoprotein subfractions and the risk of incident ulcerative colitis.

The solid red lines represent the fitted log hazard ratios [log(HR)] derived from generalized additive models (GAMs) evaluating the relative percentage of phospholipids across different lipoprotein subfractions. The shaded areas indicate the 95% confidence intervals. All models were fully adjusted for covariates in Model 3, including Age, Sex, Ethnicity, Townsend Deprivation Index, Body Mass Index, Smoking status, Alcohol use, Physical Activity, Healthy Eating Index, and Ulcerative Colitis Genetic Risk Score. In contrast to absolute concentrations, the relative percentages largely did not exhibit a consistent inverse association with the risk of ulcerative colitis.

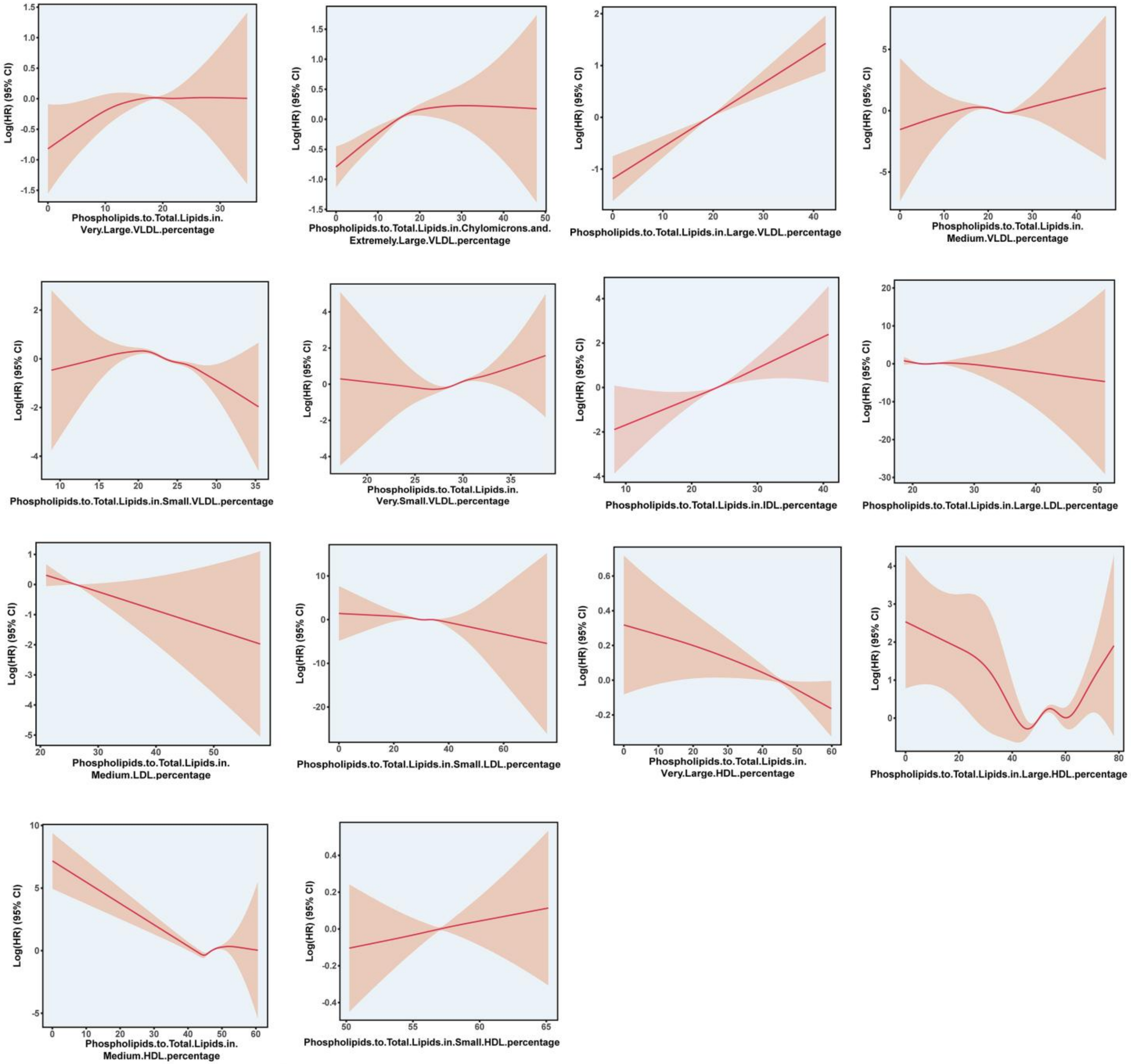
Log(HR) (95% CI)
Phospholipids.to.Total.Lipids.in.
Very.Large.VLDL.percentage
Phospholipids.to.Total.Lipids.in.Chylomicrons.and.
Extremely.Large.VLDL.percentage
Phospholipids.to.Total.Lipids.in.Large.VLDL.percentage
Phospholipids.to.Total.Lipids.in.
Medium.VLDL.percentage
Phospholipids.to.Total.Lipids.in.Small.VLDL.percentage
Phospholipids.to.Total.Lipids.in.
Very.Small.VLDL.percentage
Phospholipids.to.Total.Lipids.in.IDL.percentage
Phospholipids.to.Total.Lipids.in.Large.LDL.percentage
Phospholipids.to.Total.Lipids.in.
Medium.LDL.percentage
Phospholipids.to.Total.Lipids.in.Small.LDL.percentage
Phospholipids.to.Total.Lipids.in.
Very.Large.HDL.percentage
Phospholipids.to.Total.Lipids.in.Large.HDL.percentage
Phospholipids.to.Total.Lipids.in.
Medium.HDL.percentage
Phospholipids.to.Total.Lipids.in.Small.HDL.percentage

eFigure 2. Hexagonal binning scatter plots illustrating the correlations between plasma total phospholipids and primary lipid components.

(A) Correlation between absolute concentrations of total phospholipids and total cholesterol. **(B)** Correlation between absolute concentrations of total phospholipids and triglycerides. The density of participants is represented by the color gradient (from blue to red), where warmer colors indicate a higher concentration of overlapping data points. The solid black lines represent the fitted linear regression trends. Spearman rank correlation coefficients (*r*) and corresponding *P* values are provided for each panel, demonstrating significant positive correlations.

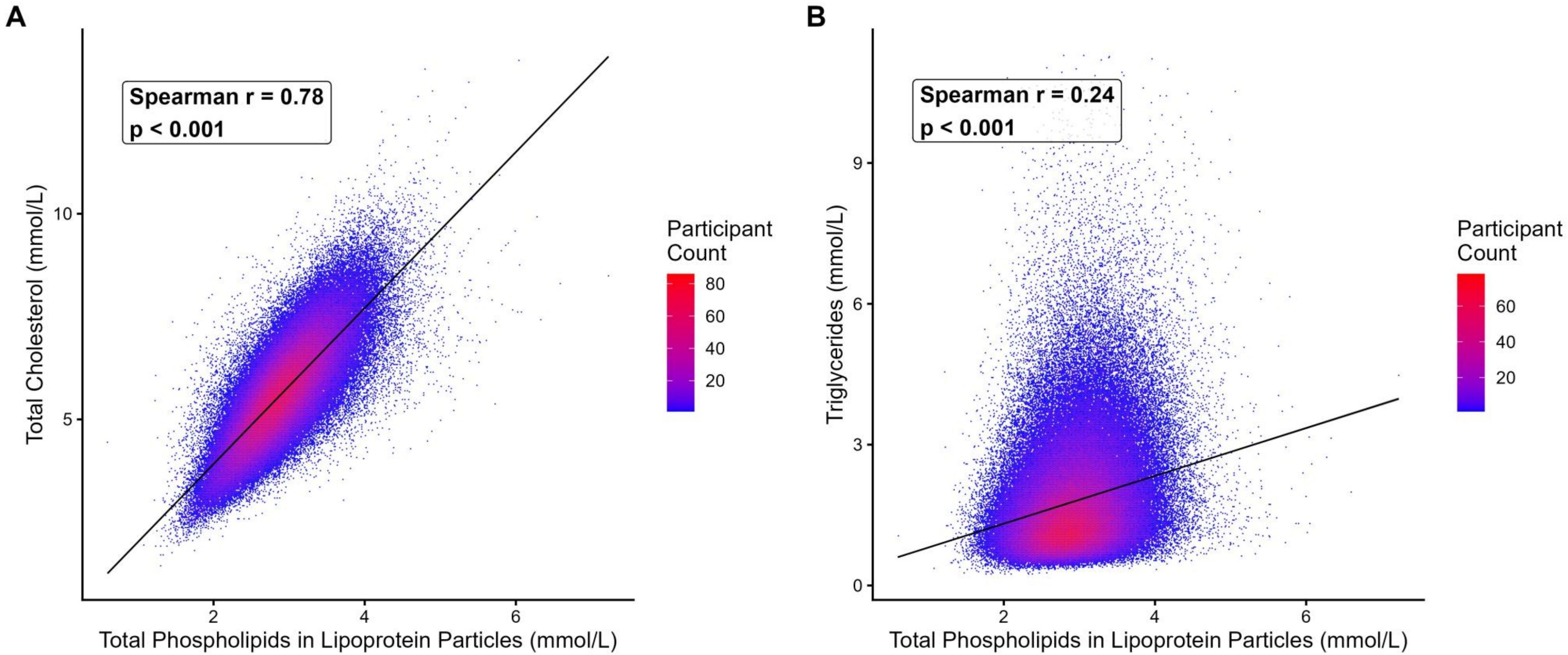

Supplement: Supplementary file 1 [file ct9-17-e01051-s001.pdf]
